# An Extract from Wax Apple (*Syzygium samarangense* (Blume) Merrill and Perry) Effects Glycogenesis and Glycolysis Pathways in Tumor Necrosis Factor-α-Treated FL83B Mouse Hepatocytes

**DOI:** 10.3390/nu5020455

**Published:** 2013-02-06

**Authors:** Szu-Chuan Shen, Wen-Chang Chang, Chiao-Li Chang

**Affiliations:** 1 Department of Human Development and Family Studies, National Taiwan Normal University, No. 162, Sec. 1, Heping East Road, Taipei 10610, Taiwan; 2 Graduate Institute of Food Science and Technology, National Taiwan University, P.O. Box 23-14, Taipei 10672, Taiwan; E-Mails: d99641001@ntu.edu.tw (W.-C.C.); carrieli700@hotmail.com (C.-L.C.)

**Keywords:** wax apple, insulin resistance, glucose metabolism, glycogenesis, glycolysis, FL83B mouse hepatocytes

## Abstract

FL83B mouse hepatocytes were treated with tumor necrosis factor-α (TNF-α) to induce insulin resistance to investigate the effect of a wax apple aqueous extract (WAE) in insulin-resistant mouse hepatocytes. The uptake of 2-[*N*-(7-nitrobenz-2-oxa-1,3-diazol-4-yl)amino]-2-deoxyglucose (2 NBDG), a fluorescent D-glucose derivative, was performed, and the metabolism of carbohydrates was evaluated by examining the expression of glycogenesis or glycolysis-related proteins in insulin-resistant hepatocytes. The results show that WAE significantly improves the uptake of glucose and enhances glycogen content in insulin-resistant FL83B mouse hepatocytes. The results from Western blot analysis also reveal that WAE increases the expression of glycogen synthase (GS), hexokinase (HXK), glucose-6-phosphate dehydrogenase (G6PD), phosphofructokinase (PFK) and aldolase in TNF-α treated cells, indicating that WAE may ameliorate glucose metabolism by promoting glycogen synthesis and the glycolysis pathways in insulin-resistant FL83B mouse hepatocytes.

## Abbreviations

TNF-α: tumor necrosis factor-α
WAE: wax apple aqueous extract
2-NBDG: 2-[*N*-(7-nitrobenz-2-oxa-1,3-diazol-4-yl)amino]-2-deoxyglucose
KRB buffer: Krebs-Ringer Bicarbonate buffer
GS: glycogen synthase
HXK: hexokinase
G6PD: glucose-6-phosphate dehydrogenase
PFK: phosphofructokinase
DM: diabetes mellitus
F12K: F12 Ham Kaighn’s
FBS: Fetal bovine serum
PBS: phosphate buffered saline
SDS: sodium dodecyl sulfate
EDTA: ethylenediamine tetraacetic acid
PMSF: phenylmethanesulfonyl fluoride
SDS-PAGE: sodium dodecyl sulfate polyacrylamide gel electrophoresis
PBST: phosphate buffer saline and Tween 20
HRP: horseradish peroxidase
ECL: enhanced chemiluminescence
G6P: glucose-6-phosphate
PP pathway: pentose phosphate pathway
NADPH: nicotinamide adenine dinucleotide phosphate reduced
ROS: reactive oxygen species
PI3K: phosphatidylinositol-3 kinase
DHAP: dihydroxyacetone phosphate.

## 1. Introduction

Diabetes mellitus (DM) is a metabolic disorder whose incidence is rapidly increasing. This chronic disease is characterized by hyperglycemia resulting from deficiencies in insulin secretion and/or insulin action [1]. Type 2 DM is the most common form of diabetes, accounting for more than 90% of cases. Insulin resistance is a characteristic feature of Type 2 DM [2].

The hyperglycemia characterizing the disease is a result of altered glucose metabolism and results in numerous complications, such as nerve and microvascular disease. The liver is an insulin-sensitive organ that regulates energy homeostasis. Liver cells have been used in an* in vitro* model to evaluate and screen antihyperglycemic agents from food ingredients [2]. In addition,* in vitro* hepatocytes retain the enzyme activities characteristic of the intact *in vivo* liver [3]; thus, they may provide a suitable model for examining liver function.

Currently, several drugs that increase insulin sensitivity are being administered clinically to ameliorate Type 2 DM. In recent years, the search for appropriate hypoglycemic agents has been focused on plants or herbs used in traditional medicine [4]. Myrtaceae plants are traditionally used to cure bronchitis, asthma, DM and inflammation by Europeans [5]. They demonstrate potent free radical scavenging, anti-oxidant, anti-mutagen and anticancer activities [6]. Wax apple (*Syzygium samarangense* (Blume) Merrill and Perry) belongs to the Myrtaceae plant family and is of economic importance in Asia and Taiwan. Wax apple fruits have reportedly demonstrated antihyperglycemic activity in alloxan-induced (Type 1 DM) diabetic mice [7]. However, studies investigating the association between wax apples and insulin resistance (Type 2 DM) are lacking. Moreover, the mechanism by which wax apples alter glucose metabolism in Type 2 DM is not clearly elucidated.

The present study aimed to investigate the effect of wax apple fruit extract (WAE) on carbohydrate metabolism in TNF-α-treated insulin-resistant FL83B mouse hepatocytes. Glucose uptake, glycogen accumulation and the expression of proteins involved in glucose metabolism were evaluated in FL83B cells. Additionally, the expressions of glycogenic and glycolytic enzymes were analyzed using Western blotting to identify the mechanisms underlying glucose metabolism in FL83B cells.

## 2. Materials and Methods

### 2.1. Chemicals and Reagents

Insulin, recombinant mouse TNF-α and F12 Ham Kaighn’s modification (F12K) medium were purchased from Sigma-Aldrich Co. (St. Louis, MO, USA). Fetal bovine serum (FBS) was obtained from Gemini Bio-Products (Woodland, CA, USA). The fluorescent dye 2-(*N*-(7-nitrobenz-2-oxa-1,3-diazol-4-yl)amino)-2-deoxyglucose (2-NBDG) was purchased from Invitrogen (Eugene, OR, USA). All of the chemicals used in this study were of analytical grade.

### 2.2. Plant Material and the Extraction, Isolation and Purification of WAE

**Figure 1:**
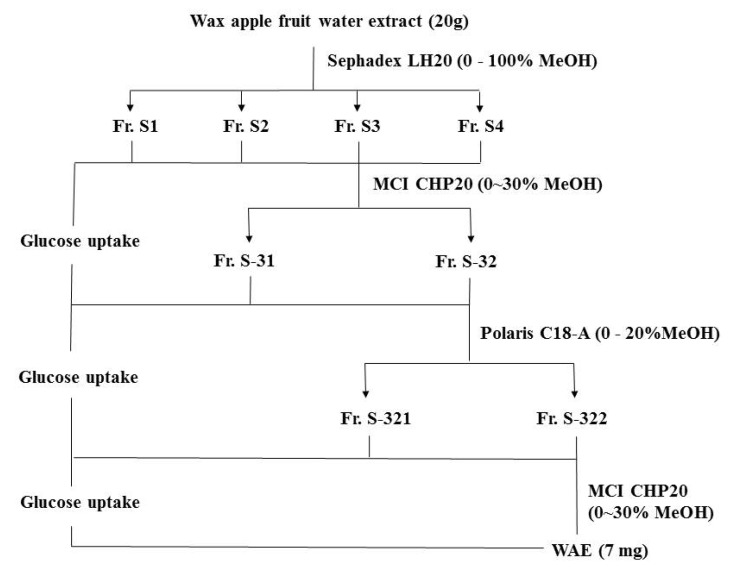
The flow chart for fractionation of wax apple fruit water extract.

The fruit of the wax apple (*Syzygium samarangense* (Blume) Merrill and Perry) was collected after the third week of blooming in 2010, July from Shuang-Hsi Township, Taipei County, Taiwan. The method of Shen *et al.* [8] was used to obtain WAE from unripe wax apple fruit water extract into a freeze-dried powder for further study (Figure 1). An aliquot of 20 g reconstituted wax apple fruit extract was run through a Sephadex LH-20 (St. Louis, MO, USA) column with 0% to 100% MeOH (500 mL) as the eluent. The fractionated eluates in the collector from single experiments were then pooled into S1, S2, S3 and S4 fractions according to the order of elution and the thin layer chromatography (TLC) profile. A silica gel precoated plate (Kieselgel 60 F254, 0.20 mm, Merck, Darmstadt, Germany) with a mobile phase of benzene:ethyl-formate:formic acid = 1:5:2 was used for TLC analysis. Fraction S3 was run through the MCI-gel CHP 20P (2 cm × 30 cm) (Mitsubishi Chemical Industries, Tokyo, Japan) using gradient elution with MeOH-H2O (0:100, 10:90, 20:80 and 30:70; 300 mL in each stage) to obtain fractions S-31 and S-32. Fraction S-32 was run through the Sephadex LH-20 column (2 cm × 30 cm) using gradient elution with H2O-MeOH to obtain fractions S-321 and S-322. Fraction S-322 was further chromatographed over an MCI-gel CHP 20P column using gradient elution with H2O-MeOH to obtain fraction A. Every 3 mL of fraction A was collected. Adjacent fractions were pooled based on the TLC profile and then freeze-dried as a powder (WAE, 7 mg). 

### 2.3. Cell Culture

The experiments were performed on mouse liver FL83B cells; a hepatocyte cell line derived from a fetal mouse (15 day to 17 day). The cells were incubated in F12K containing 10% FBS and 1% penicillin and streptomycin (Invitrogen Corporation, Camarillo, CA, USA) in 10 cm Petri dishes at 37 °C and 5% CO_2_. Experiments were performed on cells that were 80% to 90% confluent.

### 2.4. Induction of Insulin Resistance Using TNF-α and Cell Preparation

The methods were adopted from Huang *et al.* [9], with minor modifications. Briefly, the FL83B cells were seeded in 10 cm dishes and then incubated at 37 °C for 48 h to achieve 80% confluence. Serum-free F12K medium containing 20 ng/mL recombinant mouse TNF-α was then added before incubating for 5 h to induce insulin resistance. The cells were then transferred to another F12K medium containing 5 mM glucose, without (basal) or with 1000 nM insulin and 6.25 ng/mL WAE and incubated for 3 h at 37 °C. An assay of glucose uptake was then performed.

### 2.5. Determination of Glycogen

The accumulation of glycogen in FL83B cells was determined after the 3 h incubation noted in 2.4 using a glycogen assay kit (Biovision Corp., Mountain View, CA, USA). Briefly, the cells were collected, washed twice with ice-cold PBS and homogenized in 200 μL deionized water. The homogenates were boiled for 5 min to inactivate enzymes and centrifuged at 13,000× *g* for 5 min to remove the pellet. Fifty microliters of supernatant of each sample were mixed with 2 μL of Hydrolysis Enzyme Mix in a 96-well plate, and the plate was incubated at room temperature for 10 min. A 50-μL aliquot of the reaction mix (46 μL Development Buffer, 2 μL Development Enzyme Mix, 2 μL OxiRed Probe) was added to each well, and the plate was incubated at room temperature for 30 min in the dark. Absorbance at 570 nm was measured using a microplate reader (Sunrise, TECAN, Salzburg, Austria). A standard glycogen curve (0, 0.4, 0.8, 1.2, 1.6 and 2.0 μg/well) was calculated by the above method.

### 2.6. Protein Extraction from Cells

After pre-incubation in serum-free F12K medium with or without TNF-α at 37 °C for 5 h, FL83B cells were transferred to another serum-free F12K medium with/without insulin or WAE for 3 h. The medium was removed. The cells were washed twice with ice cold PBS and then lysed in ice cold lysis buffer containing 20 mM Tris-HCl (pH 7.4), 1% Triton X-100, 0.1% sodium dodecyl sulfate (SDS), 2 mM ethylenediamine tetraactic acid (EDTA), 10 mM NaF, 1 mM phenylmethanesulfonyl fluoride (PMSF), 500 μM sodium orthovanadate and 10 μg/mL antipain. Cell lysates were sonicated 4 times every 5 s with ice cooling and then centrifuged (13,000× *g*, 20 min) to recover the supernatant. The supernatant was removed as the cell extract and stored at −80 °C for further use. The protein concentration in the cell extract was determined using Bio-Rad protein assay dye reagent (Richmond, VA, USA).

### 2.7. Western Blot Analysis

Aliquots of the supernatant, each containing 50 μg protein, were used to evaluate the expression of glycogen synthase (GS), hexokinase (HXK), glucose-6-phosphate dehydrogenase (G6PD), phosphofructokinase (PFK) and aldolase. The samples were subjected to 10% sodium dodecyl sulfate polyacrylamide gel electrophoresis (SDS-PAGE). The proteins were electrotransferred to a polyvinylidene difluoride membrane. The membrane was incubated with block buffer (PBS containing 0.05% Tween-20 and 5% w/v nonfat dry milk) for 1 h, washed with PBS containing 0.05% Tween-20 (PBST) 3 times and then probed with 1:2000 diluted solutions of anti-GS (Cell Signaling Technology, Beverly, MA, USA), 1:1000 diluted solution of anti-HXK, anti-G6PD and anti-PFK antibody (Gene Tex, Irvine, CA, USA) overnight at 4 °C. The intensity of the blots probed with 1:4000 diluted solution of mouse monoclonal antibody to bind actin (Gene Tex, Irvine, CA, USA) was used as the control to ensure that a constant amount of protein was loaded into each lane of the gel. The membrane was washed 3 times for 5 min each time in phosphate buffer saline and 0.05% Tween 20 (PBST), shaken in a solution of horseradish peroxidase (HRP)-linked anti-mouse IgG or anti-rabbit IgG secondary antibody, washed a further 3 times for 5 min each time in PBST and then exposed to the enhanced chemiluminescence (ECL) reagent (Millipore) according to the manufacturer’s instructions. The films were scanned and analyzed by using UVP Biospectrum image system (Level, Cambridge, UK).

### 2.8. Statistical Analysis

The data were analyzed using one-way ANOVA and Duncan’s new multiple range tests. A *p*-value < 0.05 was considered significant.

## 3. Results

### 3.1. Effect of WAE on Glycogen Content in Insulin Resistant FL83B Mouse Hepatocytes

**Figure 2:**
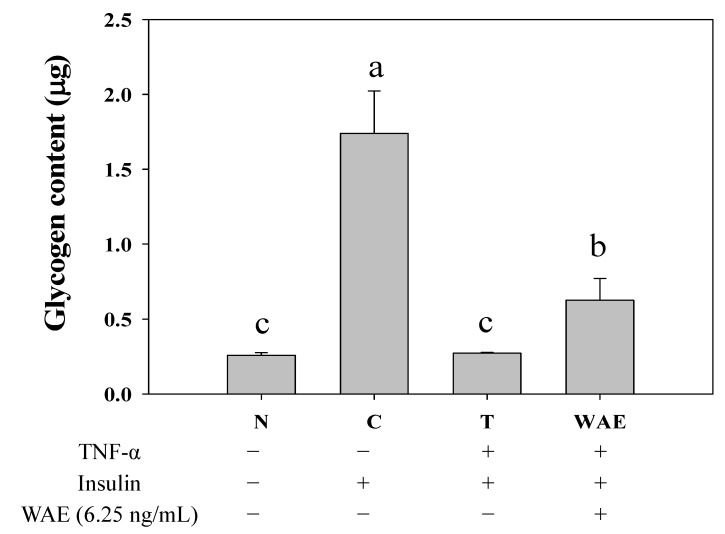
Effect of wax apple aqueous extract (WAE) on glycogen content in TNF-α-treated FL83B mouse hepatocytes. FL83B cells were incubated in serum-free F12 Ham Kaighn’s (F12K) medium, with or without added TNF-α (20ng/mL), incubated at 37 °C for 5 h, transferred to another serum-free F12K medium without (basal) or with insulin (1000nM), WAE (6.25μg/mL) and then incubated for an additional 3 h. Data of glycogen content are expressed as the mean ± SD, *n*=4. Letters a~c indicate significant differences at the 5% level. N: Normal group, cells incubated with F-12K medium. C: Control group, cells incubated with F-12K medium containing 1000nM insulin. T: TNF-α treated insulin-resistant group.

In mouse liver FL83B cells, exposure to insulin (1000 nM) for 3 h significantly increased glycogen content about 5.69-fold, from 0.26 ± 0.02 μg/mg protein in the normal group to 1.74 ± 0.28 μg/mg protein in the positive control (Figure 2). There was a 133.3% increment in glycogen in TNF-α-treated insulin-resistant cells that were treated with WAE, as well as insulin (0.63 ± 0.55 μg/mg protein), as compared with the cells treated with TNF-α, as well as insulin (0.27 ± 0.01 μg/mg protein) (Figure 2).

### 3.2. Glycogen Synthase Expression

**Figure 3:**
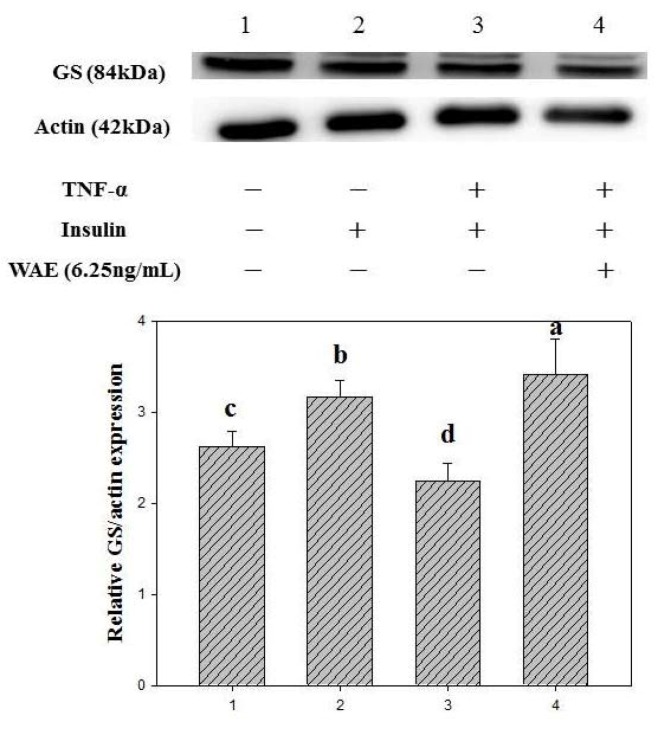
Effect of WAE on glycogen synthase expression in TNF-α-treated FL83B mouse hepatocytes. FL83B cells were incubated in serum-free F12K medium, with or without added TNF-α (20ng/mL), incubated at 37 °C for 5 h, transferred to another serum-free F12K medium with or without insulin (1000 nM), WAE (6.25μg/mL) and then incubated for an additional 30 min. The relative expressions of glycogen synthase in each treatment group were calculated using actin as the standard. Letters a~d indicate significant differences at the 5% level.

The addition of insulin alone increased the GS expression level by 20.8% in normal FL83B cells (Figure 3). The GS expression level of TNF-α-induced insulin-resistant FL83B cells decreased by 28.6% compared to that of the control group. However, WAE at a concentration of 6.25 μg/mL increased GS expression by 51.7% in insulin-resistant FL83B mouse hepatocytes (*p* < 0.05) (Figure 3).

### 3.3. Glycolysis-Related Enzyme Expression

**Figure 4:**
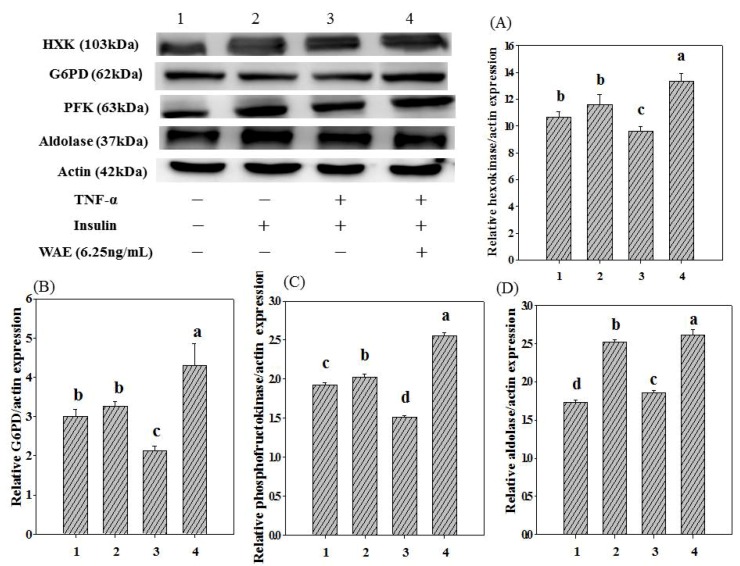
Effect of WAE on glucose metabolite-related enzymes expression in TNF-α-treated FL83B mouse hepatocytes. FL83B cells were incubated in serum-free F12K medium, with or without added TNF-α (20ng/mL), incubated at 37 °C for 5 h, transferred to another serum-free F12K medium with or without insulin (1000nM), WAE (6.25μg/mL) and then incubated for an additional 30 min. The relative expressions of hexokinase, glucose-6-phosphate dehydrogenase, phosphofructokinase and aldolase in each treatment group were calculated using actin as the standard. Letters a~d indicate significant differences at the 5% level.

Figure 4 shows the effect of WAE on glucose metabolite-related enzyme expression in TNF-α-treated FL83B cells. The results show that the expression of HXK, G6PD, PFK and aldolase in insulin alone-treated FL83B cells increased by 9.4%, 8.7%, 5.3% and 45.1%, respectively, compared to that of the normal group. By contrast, the HXK, G6PD and PFK expression levels of the TNF-α-treated FL83B cells decreased by 9.8%, 29.0% and 21.4%, respectively, compared to that of the normal group (Figure 4). However, treatment with FWFE increased expression of HXK, G6PD, PFK and aldolase by 39.2%, 101.4%, 69.5% and 40.7%, respectively, compared to the TNF-α-treated group. In addition, treatment with WAE increased expression of HXK, G6PD, PFK and aldolase by 15.7%, 31.6%, 26.7% and 4.1%, respectively, compared to the insulin alone-treated control group (Figure 4).

## 4. Discussion

The proinflammatory cytokine TNF-α plays a pivotal role in the pathogenesis of insulin resistance by the impairment of insulin signal transduction in cells and animals. The possible mechanisms for TNF-α to impair insulin signal transduction involve the downregulation of insulin receptor (IR) and insulin receptor substrate-1 (IRS-1) expressions, the inhibition of tyrosyl phosphorylation of IR and IRS-1, the increase in serine/threonine phosphorylation of IRS-1, the decrease in the activities of insulin receptor kinase and protein tyrosine phosphatases (PTPs) and the inhibition of insulin stimulated glucose transporter [9]. In our previous study, the uptake of fluorescent dye 2-NBDG in TNF-α-induced insulin-resistant FL83B cells decreased by 1.4% and 12.9%, respectively, compared to those of the normal and insulin alone-treated group. However, WAE at the concentration of 6.25 ng/mL, increased glucose uptake in TNF-α-treated FL83B mouse hepatocytes [8]. WAE might alleviate insulin resistance in TNF-α-treated FL83B cells by activating PI3K-Akt/PKB signaling and inhibiting inflammatory response via suppression of JNK, rather than ERK, activation [8]. Resurreccion-Magno *et al.* [7] investigated the hypoglycemic bioactivity of wax apple fruit in Type 1 DM mice and suggested that chalcones, an intermediate product of isoflavone biosynthesis, and polyphenol derivatives in plants and their derivates are the main anti-diabetic components. 

The liver releases glucose by hydrolyzing glycogen and uptakes glucose from the blood to maintain blood glucose homeostasis [10]. The modulation of glucose metabolism involves the performance of numerous glucose regulating enzymes in the liver [11,12]. Panneerselvam and Govindaswamy [13] found the activity of enzymes involved in glucose metabolism, including HXK, G6PD, PFK and GS, were declined in diabetic rats. GS is the primary enzyme for catalyzing glycogen synthesis in the liver. Insulin induces a series of signal transduction pathways and dephosphorylates GS, which activates this enzyme and, consequently, increases glycogen content and reduces the blood glucose level under normal conditions [14]. The metabolism of glucose can be mediated by insulin through insulin signal transduction, which stimulates the subsequent utilization of glucose and synthesis of glycogen in cells [15]. TNF-α may interfere with insulin signal transduction via phosphorylation of insulin receptors, tyrosyl phosphorylation of insulin receptor substrate-1 and activation of phosphatidylinositol-3 kinase (PI3K), therefore influencing glucose metabolism [16,17]. Recently, TNF-α has been reported to inhibit the glucose uptake ability and decrease the expression of PI3K in FL83B cells [9]. Inhibition of PI3K results in interference of gene modulation, glucose uptake and glycogen synthesis in HepG2 hepatoma cells [18]. The results from this study show that TNF-α may interfere with and cause the reduction of GS expression in the FL83B cells. WAE may increase the glycogen levels (Figure 2) and expression of GS in insulin-resistant FL83B mouse hepatocytes (Figure 3), indicating that WAE may improve insulin sensitivity, thus promoting the synthesis of glycogen in insulin-resistant FL83B mouse hepatocytes.

Most cells in the body are dependent upon a continuous supply of glucose to supply energy in the form of ATP [19]. Glycolysis is triggered when glucose is transported into cells. In a Type 2 DM rats model, the activity of HXK is significantly decreased [20]. HXK is a key enzyme for the first step of glycolysis that catalyzes phosphorylation of glucose into glucose-6-phosphate (G6P). Insulin has been reported to increase the activity of HXK and enhance glucose utilization in muscle cells [21]. Treatment with TNF-α decreased the HXK expression level in normal FL83B cells. However, WAE increased the HXK expression level in insulin-resistant FL83B mouse hepatocytes (Figure 4A), indicating that WAE may enhance the glycolysis pathway in insulin-resistant FL83B mouse hepatocytes.

G6PD is the rate determining step key enzyme in the pentose phosphate pathway (PP pathway) that catalyzes glucose-6-phosphate to produce 6-phosphogluconolactone, as well as nicotinamide adenine dinucleotide phosphate reduced (NADPH) [22]. Environmental stresses, such as drugs, inflammatory reactions, UV exposure, ion radiation and oxidative chemicals, may lead to the production of substantial amounts of reactive oxygen species (ROS) and high oxidative stress and, subsequently, inhibit G6PD activity in the PP pathway [23]. TNF-α triggers the production of ROS and depresses the activity of G6PD in cells [24]. Insulin promotes the production of NADPH catalyzed by G6PD and increases the anti-oxidative capacity in hepatocytes and adipocytes. The activity of G6PD is also modulated by the phosphatidylinositol-3 kinase (PI3K) pathway in insulin signaling cascades, which simultaneously regulates glucose metabolism [25]. In this study, WAE significantly increased the expression of G6PD in insulin-resistant FL83B hepatocytes compared to results in the control group (Figure 4B), indicating that WAE may promote cells to go through the pentose phosphate pathway for glucose metabolism.

In contrast to glucose, fructose is entirely metabolized in the liver. PFK is the rate determining enzyme in the first step of fructose metabolism. PFK catalyzes the phosphorylation of C-1 in fructose-6-phosphate, causing the irreversible formation of fructose-1,6-bisphosphate and promoting glycolysis. Strack [26] reported that the activity of PFK significantly decreased in tissues, including liver, muscles and adipose of streptozotocin-induced diabetic rats. However, treatment with metformin normalized the PFK activity in muscles and adipocytes, but partially restored PFK activity in hepatocytes of those diabetic rats. It may be that the long-term exposure to low glucose concentrations irreversibly inactivates glucose metabolic enzymes in liver and decreases the efficiency of glycolysis and glycogenesis as a consequence [26]. The activity of PFK may be regulated through cytokines or insulin secreted from cells [27]. Insulin increases the production of fructose-2,6-bisphosphate, a PFK activating factor, and activates PFK [28]. Aspirin has been recognized to effectively restore glucose metabolism by repairing the quaternary structure of PFK in diabetic rats [29]. The results from this study show that WAE increased the expression of PFK in insulin-resistant FL83B (Figure 4C), suggesting that WAE may provide a similar effect to aspirin in DM patients.

Aldolase is an essential enzyme in glycolysis, which catalyzes hexose bisphosphates (*i.e.*, fructose-1,6-bisphosphate) decomposition into triose phosphates, including glyceraldehyde-3-phosphate and dihydroxyacetone phosphate (DHAP) via a reversible aldol condensation reaction [30]. The activity of aldolase has been reported to be affected by cytokines and various environmental stresses [31]. The results from this study show that TNF-α suppresses aldolase expression; however, WAE increases the aldolase expression of TNF-α treated cells (Figure 4D), indicating that WAE may alleviate the damage from free radicals, such as oxidative stress or ROS, of hepatocytes caused by TNF-α, thereby improving the metabolism of hepatic glucose.

## 5. Conclusions

The present study investigated the effects of WAE on the metabolism of carbohydrates in TNF-α-induced insulin-resistant FL83B mouse hepatocytes. The results show that WAE improves glucose uptake in TNF-α-treated FL83B cells. Furthermore, WAE increases expression of GS, HXK, G6PD, PFK and aldolase, suggesting increased glycolysis and gluconeogenesis, and WAE increases glycogen storage. These findings suggest that wax apple fruit may mitigate the hyperglycemia in Type 2 DM patients; therefore, it has the potential to be developed into a functional food or dietary supplement that prevents and/or alleviates DM. Further investigation on the purification and identification of active compounds in WAE is currently underway in our laboratory.

## References

[1] Roa B.K., Sudarshan P.R., Rajasekhar M.D., Nagaraju N., Roa C.A. (2003). Antidiabetic activity of *Terminalia pallida* fruit in alloxan induced diabetic rats. J. Ethnopharmacol..

[2] Cheng H.L., Huang H.K., Chang C.I., Tsai C.P., Chou C.H. (2008). A cell-based screening identifies compounds from the stem of *Momordica charantia* that overcome insulin resistance and activate AMP activated protein kinase. J. Agric. Food Chem..

[3] Hengstler J.G., Utesch D., Steinberg P., Platt K.L., Diener B., Ringel M., Swales N., Fischer T., Biefang K., Gerl M., Böttger T., Oesch F. (2000). Cryopreserved primary hepatocytes as a constantly available *in vitro* model for the evaluation of human and animal drug metabolism and enzyme induction. Drug Metab. Rev..

[4] Rates S.M. (2001). Plants as source of drugs. Toxicon.

[5] Gurib-Fakim A. (1996). Phytochemical screening of 38 Mauritian medicinal plants. Rev. Agric. Sucr. Ile Maurice.

[6] Neergheen V., Soobrattee M., Bahorun T., Aruoma O. (2006). Characterization of the phenolic constituents in *Mauritian endemic* plants as determinants of their antioxidant activities *in vitro*. J. Plant Physiol..

[7] Resurreccion-Magno M., Villasenor I., Harada N., Monde K. (2005). Antihyperglycaemic flavonoids from *Syzygium samarangense* (Blume) Merr. and Perry. Phytother. Res..

[8] Shen S.C., Chang W.C., Chang C.L. (2012). Fraction from wax apple [*Syzygium samarangense* (Blume) Merrill and Perry] fruit extract ameliorates insulin resistance via modulating insulin signaling and inflammation pathway in tumor necrosis factor α-treated FL83B mouse hepatocytes. Int. J. Mol. Sci..

[9] Huang D.W., Shen S.C., Wu J.S.B. (2009). Effects of caffeic acid and cinnamic acid on glucose uptake in insulin-resistant mouse hepatocytes. J. Agric. Food Chem..

[10] Kim H.P., Son K.H., Chang H.W., Kang S.S. (2004). Anti-inflammatory plant flavonoids and cellular action mechanism. J. Pharmacol. Sci..

[11] Ferrer J.C., Favre C., Gomis R.R., Fernandez-Novell J.M., Garica-Rocha M., de la Iglesia N., Cid E., Guinovart J.J. (2003). Control of glycogen deposition. FEBS Lett..

[12] Iynedjian P.B. (2009). Molecular physiology of mammalian glucokinase. Cell. Mol. Life Sci..

[13] Panneerselvam R.S., Govindaswamy S. (2002). Effect of sodium molybdate on carbohydrate metabolizing enzymes in alloxan-induced diabetic rat. J. Nutr. Biochem..

[14] Saltiel A.R., Kahn C.R. (2001). Insulin signaling and the regulation of glucose and lipid metabolism. Nature.

[15] Zick Y. (2001). Insulin resistance: A phosphorylation-based uncoupling of insulin signaling. Trends Cell Biol..

[16] Cheng J.T., Liu I.M. (2000). Stimulatory effect of caffeic acid on α1A-adrenoceptors to increase glucose uptake into cultured C2C12 cells. Naunyn-Schmiedeberg’s Arch. Pharmacol..

[17] Cichy S.B., Uddin S., Danilkovich A., Guo S., Klippel A., Unterman T.G. (1998). Protein kinase B/Akt mediates effect of insulin on hepatic insulin-like growth factor-binding protein-1 gene expression through a conserved insulin response sequence. J. Biol. Chem..

[18] González-Espinosa C., Romero-Ávila M.T., Mora-Rodríguez D.M., González-Espinosa D., García-Sáinz J.A. (2001). Molecular cloning and functional expression of the guinea pig α1A-adrenoceptor. Eur. J. Pharmacol..

[19] Gropper S.S., Smith J.L., Groff J.L. (2009). Advanced Nutrition and Human Metabolism.

[20] Clore J.N., Stillman J., Sugerman H. (2000). Glucose-6-phosphate flux *in vitro* is increased in type 2 diabetes. Diabetes.

[21] Ivy J.L., Sherman W.M., Cuyler C.L., Katz A.L. (1986). Exercise and diet reduce muscle insulin resistance in obese Zucker rat. Am. J. Physiol..

[22] Abdel-Rahim E.A., El-Saadany S.S., Abo-Eytta A.M., Wasif M.M. (1992). The effect of sammo administration on some fundamental enzymes of pentose phosphate pathway and energy metabolities of alloxanized rats. Nahrung.

[23] Nikolaidis M.G., Jamurtas A.Z., Paschalis V., Kostaropoulos I.A., Kladi-Skandali A., Balamitsi V., Koutedakis Y., Kouretas D. (2006). Exercise-induced oxidative stress in G6PD-deficient individuals. Med. Sci. Sports Exerc..

[24] Ho H.Y., Cheng M.L., Chiu D.T. (2007). Glucose-6-phosphate dehydrogenase-from oxidative stress to cellular functions and degenerative diseases. Redox. Rep..

[25] Wagle A., Jivraj S., Garlock G.L., Stapleton S.R. (1998). Insulin regulation of glucose-6-phosphate dehydrogenase gene expression is rapamycin-sensitive and requires phosphatidylinositol 3-kinase. J. Biol. Chem..

[26] Strack T. (2008). Genetics and molecular biology protein kinase C-[zeta] as an AMP-activated protein kinase kinase kinase: The protein kinase C-[zeta]-LKB1-AMP-activated protein kinase pathway. Drugs Today (Barc.).

[27] Silva D.D., Zancan P., Coelho W.S., Gomez L.S., Sola-Penna M. (2010). Metformin reverses hexokinase and 6-phosphofructo-1-kinase inhibition in skeletal muscle, liver and adipose tissue from streptozotocin-induced diabetic mouse. Arch. Biochem. Biophys..

[28] Deprez J., Vertommen D., Alessi D.R., Hue L., Rider M.H. (1997). Phosphorylation and activation of heart 6-phosphofructo-2-kinase by protein kinase B and other protein kinases of the insulin signaling cascades. J. Biol. Chem..

[29] Spitz G.A., Furtado C.M., Sola-Penna M., Zancan P. (2009). Acetylsalicylic acid and salicylic acid decrease tumor cell viability and glucose metabolism modulating 6-phosphofructo-1-kinase structure and activity. Biochem. Pharmacol..

[30] Yamakoshi Y., Nagano T., Hu J.C., Yamakoshi F., Simmer J.P. (2011). Porcine dentin sialoprotein glycosylation and glycosaminoglycan attachments. BMC Biochem..

[31] Huang Y., Shinzawa H., Togashi H., Takahashi T., Kuzumaki T., Otsu K., Ishikawa K. (1995). Interleukin-6 down regulates expression of the aldolase B and albumin genes through a pathway involving the activation of tyrosine kinase. Arch. Biochem. Biophys..

